# Recurrent Aneurysmal Bone Cyst of the Distal Fibula Treated with Denosumab and Curettage

**DOI:** 10.1155/2018/1574343

**Published:** 2018-12-09

**Authors:** Philip B. Fontenot, Jose Jesurajan, Marilyn Bui, Damon Reed, Odion Binitie

**Affiliations:** ^1^Department of Orthopaedic Surgery, University of South Florida, Tampa, FL, USA; ^2^Department of Pathology, Moffitt Cancer Center, Tampa, FL, USA; ^3^Department of Sarcoma, Moffitt Cancer Center, Tampa, FL, USA

## Abstract

We report the case of a 13-year-old girl with multiple recurrences of an aneurysmal bone cyst of the distal fibula successfully treated with denosumab and curettage. Aneurysmal bone cysts are locally aggressive lesions with high rates of recurrence. The novel use of denosumab with curettage in a long bone showed a favorable outcome with no adverse events or signs of recurrence three years after treatment.

## 1. Introduction

Aneurysmal bone cysts (ABCs) are rare, benign, locally aggressive lesions that occur most frequently in the first or second decade of life [1]. The prevalence of ABCs is 0.14 cases per 100,000 individuals, and they constitute approximately 1% of all bone tumors [1]. The most common sites include the vertebral bodies and long bones, particularly the metaphysis of the distal femur or proximal tibia [2]. ABCs were initially thought to be caused by vascular disturbances in the bone leading to increased intraosseous pressure which caused local destruction and distension [3]. More recent evidence shows a genetic component, specifically, a translocation-induced upregulation of the ubiquitin-specific protease USP6 (Tre2) gene, which favors a primary neoplastic process rather than a mechanical or vascular one [4]. The majority are considered primary lesions; however, up to 30% can be considered secondary tumors arising from chondroblastomas, osteoblastomas, nonossifying fibromas, and other benign osseous lesions [1].

Patients typically present after a nontraumatic episode with complaints of pain, swelling, or the presence of a palpable mass [1]. Radiographic imaging shows an expansile, eccentric, lytic lesion with thin cortices and septae present. Cross-sectional MRI may reveal septations, contrast enhancement with edema, and “fluid-fluid levels” which correspond to a demarcation of blood with different densities [5].

Treatment generally consists of intralesional curetting and bone grafting with or without adjuvant therapy. Wide resection is considered for lesions that have destroyed the bone, while for large tumors in the axial skeleton or pelvis, embolization and sclerotherapy have been utilized. All of these modalities have associated morbidity and risk of complications. Curettage with or without bone grafting has been shown to have a highly variable recurrence rate of anywhere from 7 to 30% in larger studies [2, 6]. Younger age, open physes, increase in mitotic figures, and increased cellularity have all been implicated in the high rate of local recurrence [7, 8]. Denosumab, a monoclonal antibody to the RANK ligand, has recently been used in a few cases and has been shown to decrease tumor burden size, reduce pain, resolve associated neurologic symptoms, and show evidence of radiographic healing [9–11]. In this report, we discuss a specific case of an ABC with multiple recurrences in a young patient treated with denosumab followed by curettage and cementation who is recurrence free three years later, without complications. The patient and her mother provided consent for publication.

## 2. Case Report

**Figure 1:**
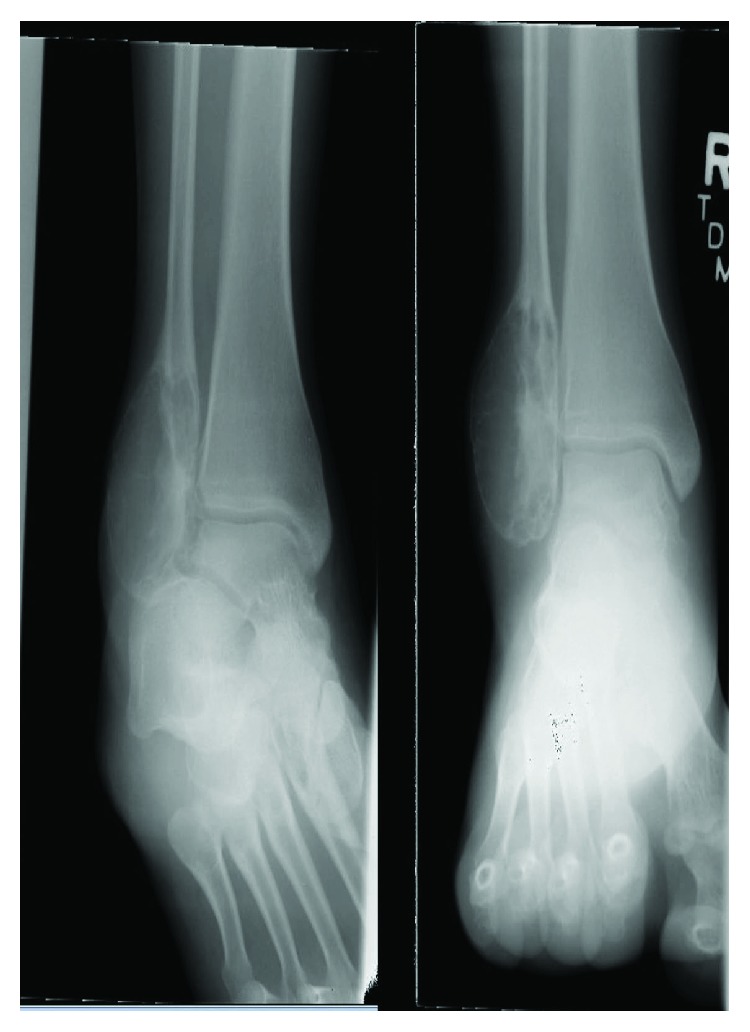
AP and mortise radiographs of the ankle demonstrating a lytic, expansile, geographic bone lesion with no cortical disruption.

**Figure 2:**
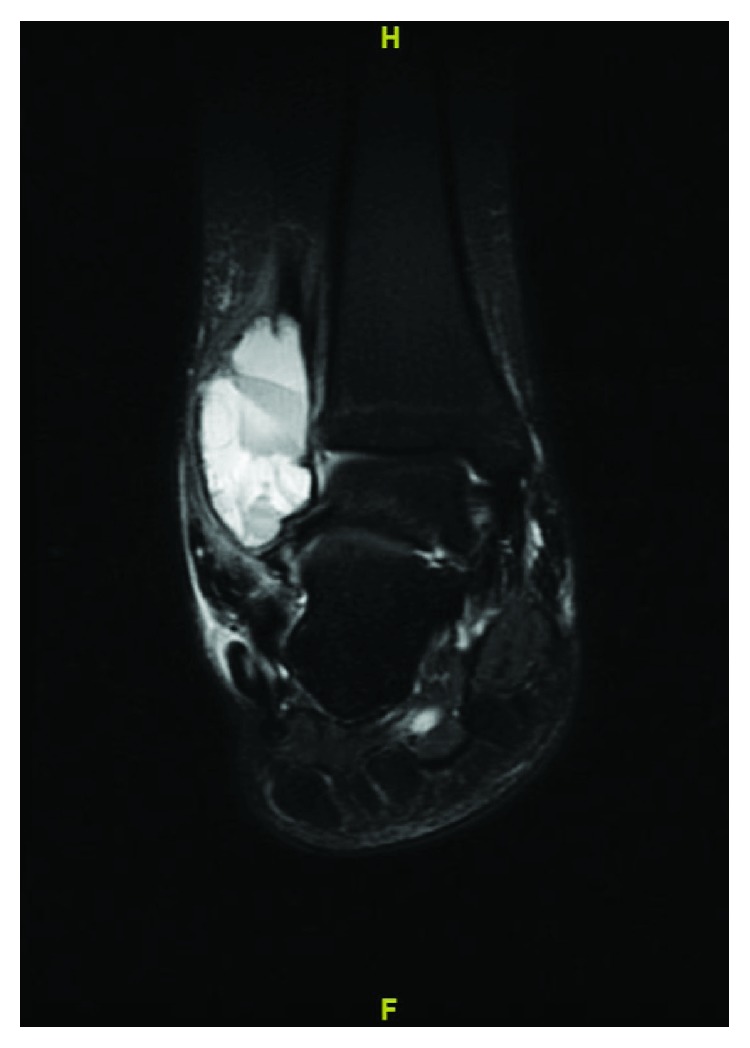
STIR (short tau inversion recovery) weighted MRI of the ankle showing 5 × 5.2 × 3.6 cm multiloculated, expansile lesion with fluid-fluid levels.

**Figure 3:**
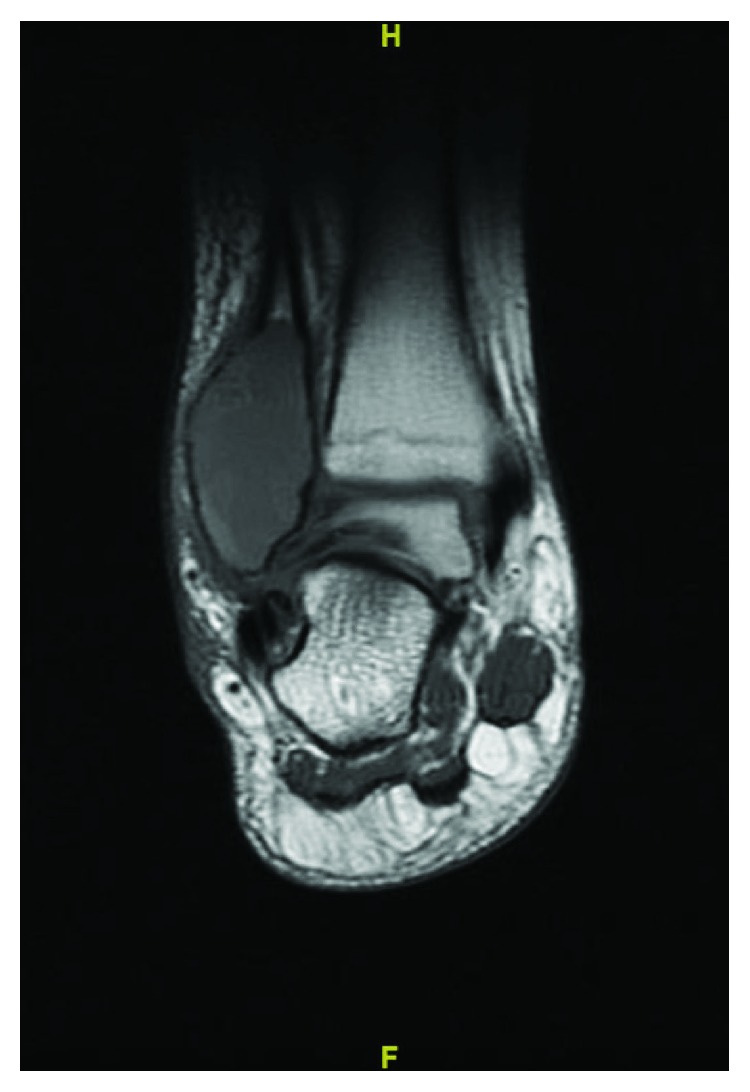
T1 weighted MRI of the ankle showing 5 × 5.2 × 3.6 cm multiloculated, slightly hyperintense compared to surrounding muscle expansile lesion.

A 13-year-old female initially presented with a history of pain and swelling along her right distal fibula. A physical exam was remarkable for point tenderness along the distal fibula with no skin changes and an otherwise normal neurovascular examination. Radiographs obtained demonstrated a lytic, expansile, geographic lesion with no cortical disruption or periosteal reaction (Figure 1). MRI revealed a multiloculated, minimally enhancing, expansile lesion (Figures 2 and 3).

**Figure 4:**
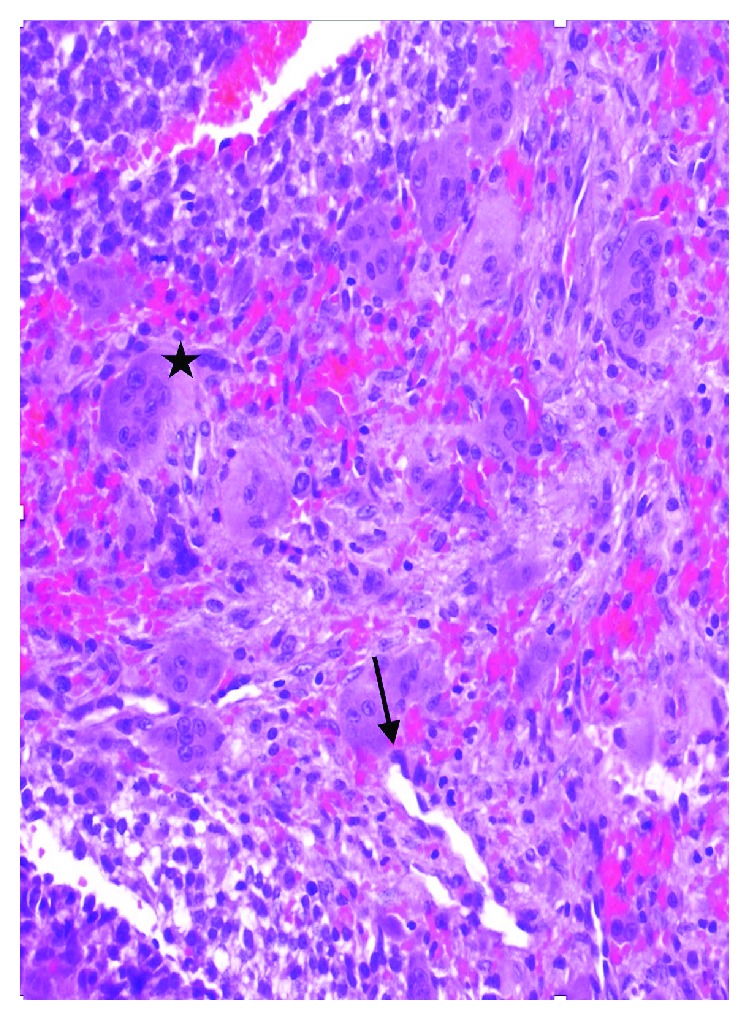
Photomicrographs demonstrating the wall of an ABC partially lined by proliferation of bland fibroblasts (Black arrow) with underlying osteoclast-type giant cells (star) and blood (hematoxylin and eosin, ×20).

**Figure 5:**
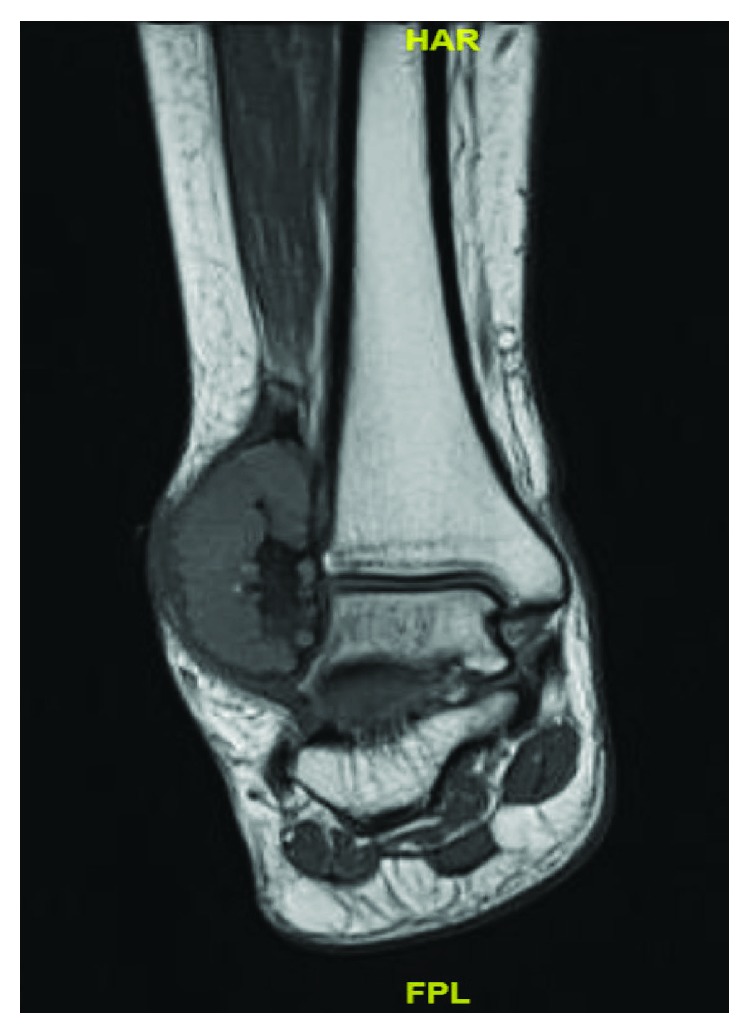
T1 weighted MRI showing a homogeneously slightly hyperintense to muscle lobulated lesion with multiple fluid-fluid levels.

**Figure 6:**
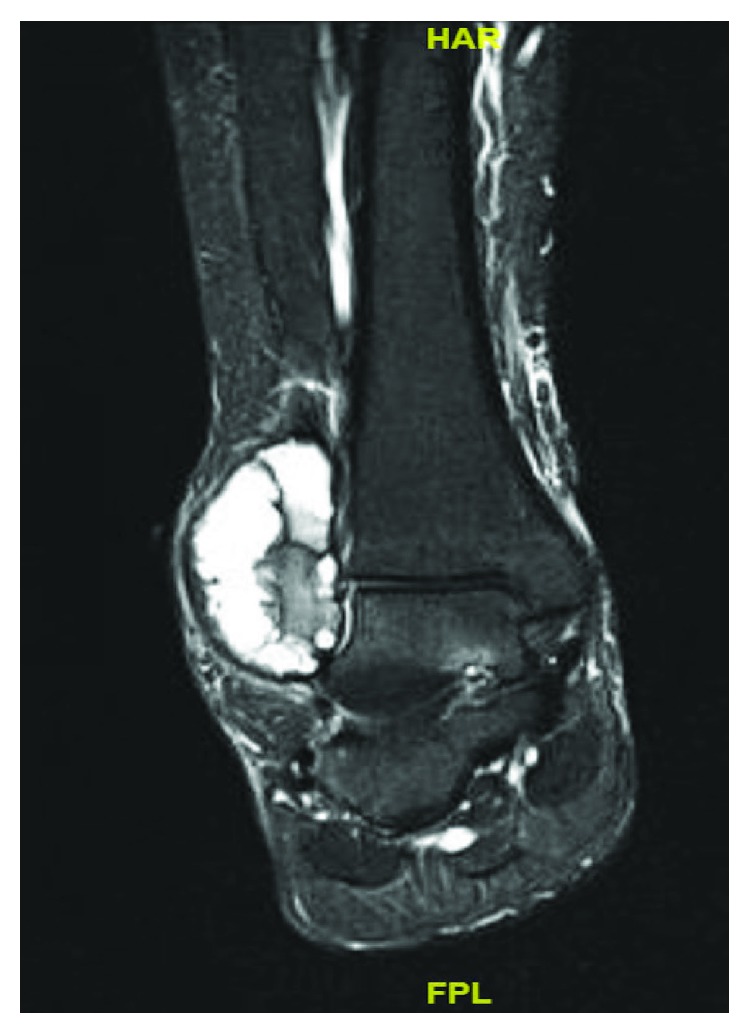
STIR (short tau inversion recovery) weighted MRI showing a homogeneously lobulated, hyperintense lesion with multiple fluid-fluid levels.

She underwent an open biopsy which showed cyst-like spaces filled with blood surrounded by fibrous septa composed of a cellular proliferation of bland fibroblasts, scattered multinucleated giant cells, and chronic inflammation consistent with the diagnosis of an ABC (Figure 4). A curettage, application of phenol, and allograft bone grafting were performed, and she was followed with serial radiographs. Six months following surgery, she presented to the clinic complaining of pain with sprinting. Radiographs obtained showed resorption of the bone graft indicating local recurrence. MRI showed a homogeneously lobulated, hyperintense lesion with multiple fluid-fluid levels confirming the recurrence (Figures 5 and 6). She underwent a repeat extended curettage with the application of phenol and bone grafting at about one year following her index procedure.

**Figure 7:**
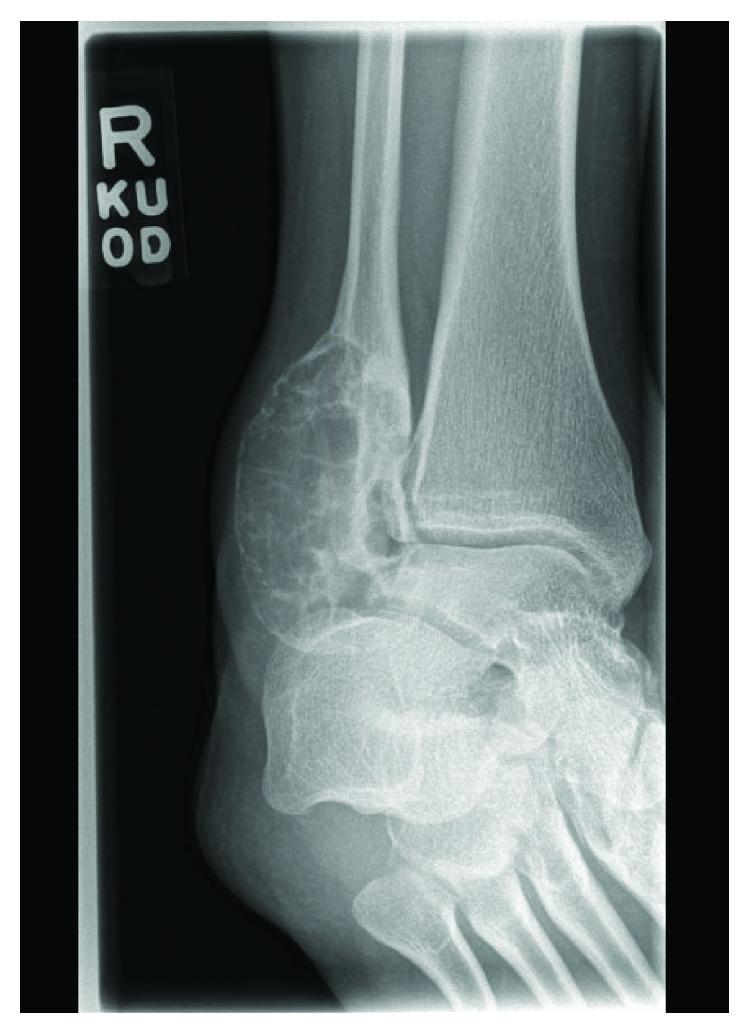
Pretreatment with denosumab: mortise radiograph showing a large lytic, expansile bone lesion with cortical disruption.

**Figure 8:**
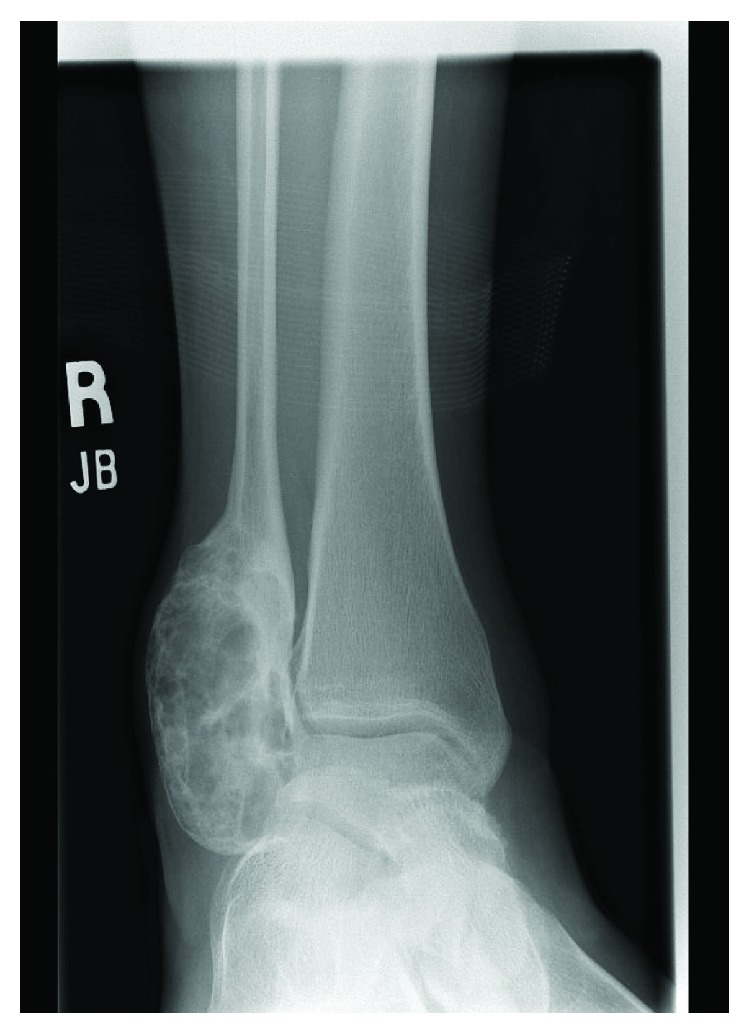
Posttreatment with denosumab after 1 year shows increased mineralization and no further expansion or cortical disruption.

Approximately eleven months following her second procedure and eighteen months following her index procedure, she again complained of pain in the right ankle with exercise. Repeat radiographs demonstrated another recurrence. Since she had failed two operative treatments, a referral to pediatric oncology was placed for the consideration of denosumab therapy for off-label use. The patient and her mother elected to proceed with the initiation of therapy after the risks including osteonecrosis of the jaw, and hypocalcemia had been discussed. The regimen used to treat giant cell tumor (GCT) was implemented which consisted of subcutaneous denosumab (120 mg) given every 4 weeks (with additional 120 mg SC doses on days 8 and 15 in cycle 1 only) for a total of 12 months [12]. She was administered vitamin D and calcium supplements daily and 3 months after the last denosumab dose to mitigate her risk of hypocalcemia, and labs were checked prior to each injection. Her pain improved following the initiation of treatment, and she was asymptomatic at completion of therapy. Serial radiographs were obtained throughout her treatment which showed increased mineralization and no further expansion of her lesion throughout the course of denosumab therapy (Figures 7 and 8).

**Figure 9:**
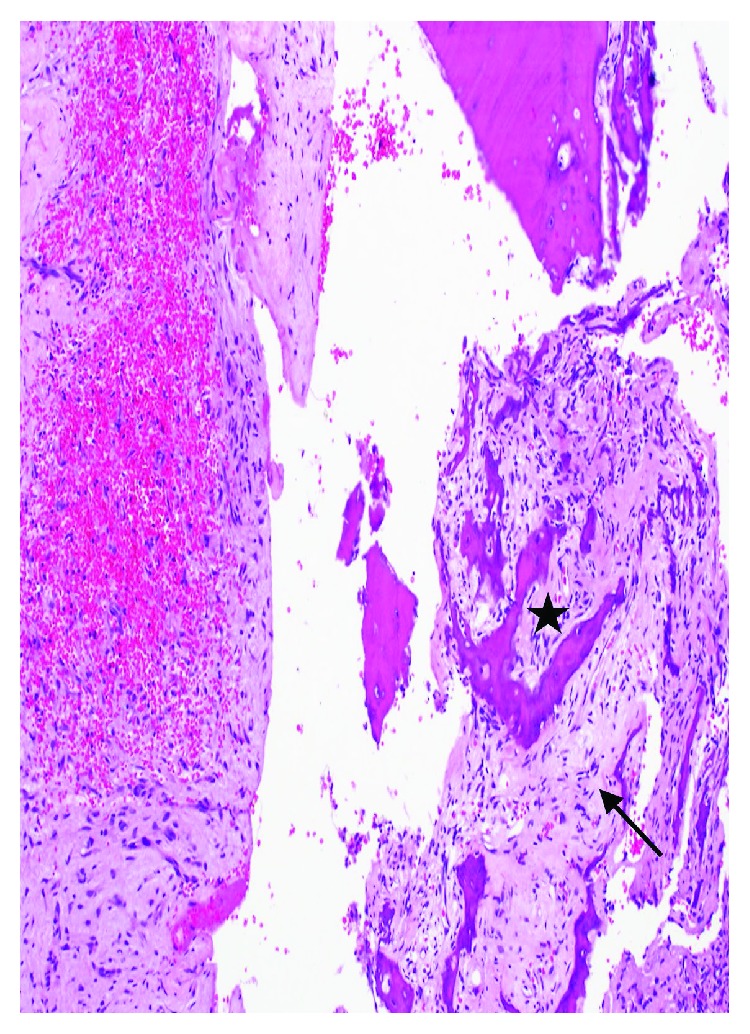
Photomicrographs taken after denosumab treatment demonstrating fibrous connective tissue with bland-looking spindle cells (arrow), focal hemorrhage, and reactive bone formation (star) admixed with scattered inflammatory cells; multinucleated giant cells are absent.

**Figure 10:**
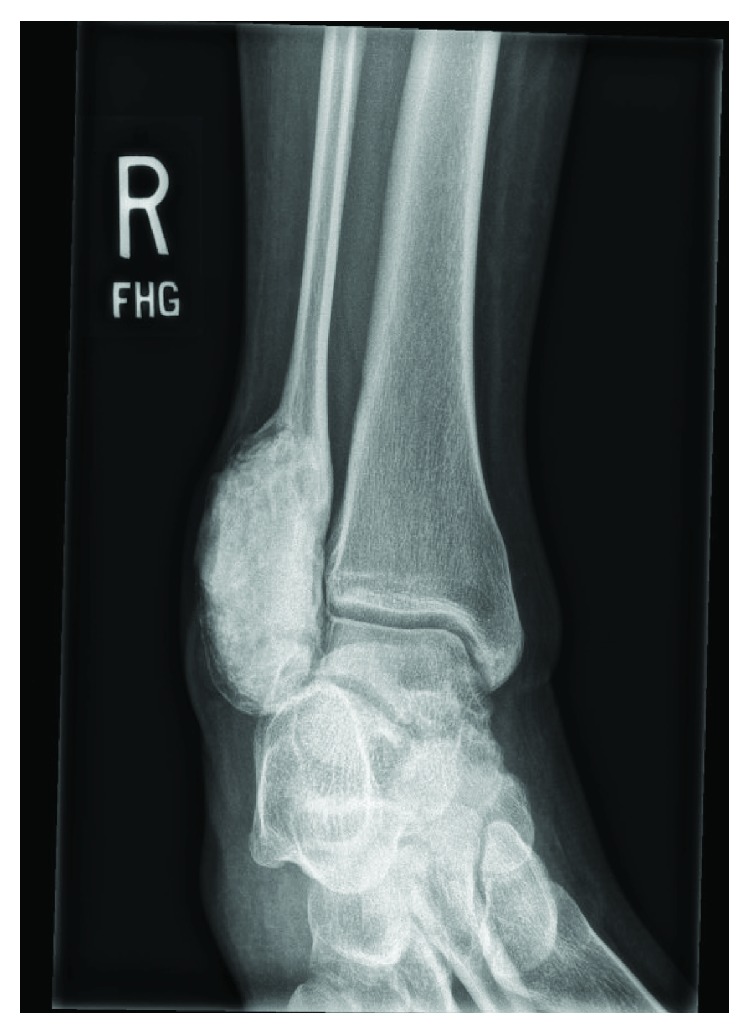
Mortise radiographs taken at 10 months following denosumab treatment and surgery demonstrating no local recurrence.

**Figure 11:**
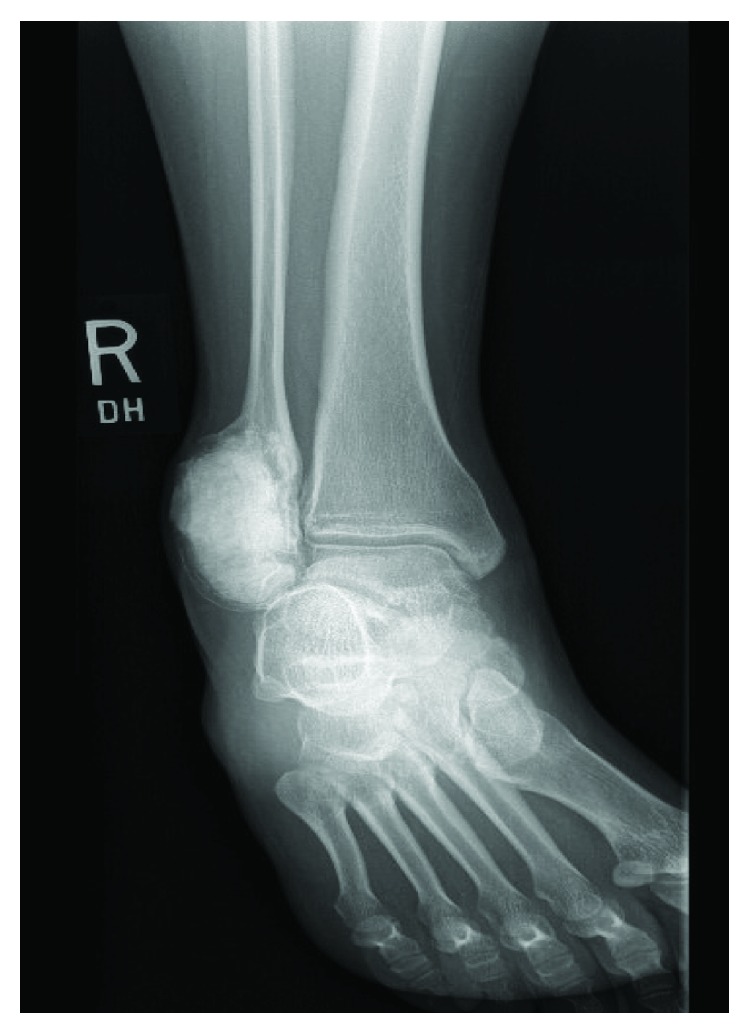
Mortise radiographs taken at 3 years following denosumab treatment and surgery demonstrating no local recurrence.

One year following the initiation of denosumab therapy, she underwent open biopsy, curettage with high-speed burring, and cement augmentation. The lesion was filled with thickened calcifications and septae. Her histopathology at the time of surgery showed numerous fragmented bones admixed with fragments of fibrous connective tissue with bland fibroblasts with no significant component of multinucleated giant cells present (Figure 9). She has been followed for three years with clinical and radiographic examination, has resumed all normal activity without pain, and has had no recurrence of her disease (Figures 10 and 11).

## 3. Discussion

The term “aneurysmal bone cyst” was first coined in 1942 by Dr. Jaffe and Dr. Lichenstein when describing lesions in the spine and pelvis [13]. The treatment at that time, curettage and bone grafting to fill the void, is still widely used today. Modifications have been made since this initial description including the use of a high-speed burr which showed a 12% local recurrence rate in extremity [7]. Another study in the spine showed no incidence of recurrence in eight patients using the same surgical technique [14]. Although good outcomes following curettage and bone grafting have been achieved, the recurrence rate following treatment is still significantly high as seen in our patient and noted in the literature [2, 6]. This has led some authors to use adjuvant therapy such as phenol in conjunction with curettage and bone grafting. Phenol is thought to necrotize or “wash” residual tumor cells following curettage and has shown a recurrence rate of 7% compared to 41% when used with curettage and bone grafting alone in simple bone lesions [15]. Using phenol to treat ABCs, Bitzan et al. demonstrated no recurrences in 9 cases after surgery with curettage, phenol, and bone grafting [16]. En bloc resection has the lowest risk of recurrence; however, the morbidity depending on the location is much higher.

Newer, pharmacologic treatments have recently been evaluated in hopes to limit the morbidity associated with surgery. The role of denosumab, a receptor activator of nuclear kappa B ligand (RANKL) inhibitor, has been studied for the treatment of benign bone tumors including giant cell tumor (GCT) as well as for metastatic bone disease. The RANK signaling pathway is essential for skeletal homeostasis, and disruption of this pathway can cause increased bone resorption. In a normal tissue, osteoblasts secrete RANKL which binds to the RANK receptor on osteoclasts causing increased bone resorption [17]. In GCT, neoplastic stromal cells express high concentrations of RANKL and activate RANK on osteoclast-like giant cells leading to increased bone destruction and osteolysis [18]. This interaction of increased stimulation of RANKL/RANK has also been linked to the pathophysiology seen in ABC in recent studies [10, 19]. Denosumab is approved for the treatment of osteoporosis, multiple myeloma, skeletal metastasis, and giant cell tumor of bone (GCT) [20]. A recent clinical trial evaluating the safety and efficacy of denosumab treatment for GCT showed promising results with fewer patients undergoing surgery after treatment and those who did undergo surgery, having a less morbid procedure than previously planned [21].

There have been several case reports highlighting the efficacy of denosumab in the spine and sacrum, but very few have reported on outcomes in long bones. Using denosumab for a recurrent ABC of the distal radius, Pauli et al. [22] were able to consolidate a locally aggressive and destructive lesion into a resectable lesion and implement limb-preserving surgery in an otherwise unresectable lesion. More recently, two cases of ABC in long bones, one of which was recurrent, have been treated with denosumab for an average of 7 months followed by curettage and bone grafting with histologic compete response to treatment with a median of 23-month follow-up [22]. Kurucu et al. [23] treated nine cases of ABC with denosumab for an average of 12 months, of which two eventually needed surgery. Clinical symptoms including pain and limping regressed completely in 3 months, and eight out of nine lesions decreased in size and number of cysts. At the time of treatment for our patient, we mirrored the typical length of time for denosumab treatment for GCT, which was close to a year. However, current trends are for treatment lasting 3-4 months prior to surgical intervention for GCT. We chose to present this case given the over 3-year follow-up which is longer than previously reported in [12, 22, 23]. Although segments of the fibula are resectable, resection of the distal fibula can lead to lifelong ankle instability, even with reconstruction, so we chose to preserve it.

The emerging safety data in the pediatric population should be considered when selecting denosumab and providing informed consent to patients and families. Different from adults and seemingly rather common in patients under 10 years of age, rebound hypercalcemia has been reported 3-4 months following the last dose of denosumab [23, 24]. While an upper age limit has not been determined, this may correlate with skeletal maturity and there are few published reports in teens [20, 23]. Our patient was 14 at the start of her denosumab treatment, close to the average age of female skeletal maturity. Furthermore, her radiographs showed closing growth plates. We did not observe symptoms consistent with hypercalcemia in this patient who was close to skeletal maturity.

## 4. Conclusion

Our findings support the use of denosumab for those lesions that are locally aggressive or unresectable due to location or in patients with failed primary treatment and multiple recurrences. The utilization of cement, in addition to providing immediate structural support in this now skeletally mature patient, may have also added additional adjuvant treatment to the lesion with its exothermic reaction during curing. We were able to preserve limb and function, and our patient tolerated denosumab very well. Considerations for this therapy include the risk of osteonecrosis of the jaw, the need for daily calcium supplementation, and it being contraindicated in pregnancy. It remains unclear if denosumab would decrease the risk of recurrence in a population of patients with ABC. Further studies evaluating long-term results with more cases are needed to better estimate efficacy of the use of denosumab for complicated ABCs not amenable to resection or in cases responding to surgery alone.
